# The Sleep Quality of the Frontline Healthcare Workers and the Improving Effect of Tai Chi

**DOI:** 10.3389/fpsyt.2022.883590

**Published:** 2022-05-02

**Authors:** Jingye Zhan, Kangdi Yang, Zhuoer Sun, Lingling Bai, Xiaoying Lu, Xiuhong Wang, Weizhi Liu, Chen Yi, Lina Wang

**Affiliations:** ^1^Lab for Post-traumatic Stress Disorder, Faculty of Psychology and Mental Health, Naval Medical University, Shanghai, China; ^2^Department of Traditional Chinese Medicine, Naval Medical University, Shanghai, China; ^3^Department of Nursing, Changhai Hospital, Naval Medical University, Shanghai, China; ^4^Department of Infectious Diseases, Changhai Hospital, Naval Medical University, Shanghai, China

**Keywords:** sleep quality, Tai Chi, frontline healthcare workers, COVID-19, anxiety

## Abstract

**Background:**

A number of studies have documented that coronavirus disease 2019 (COVID-19) brought more negative impact on the physical and psychological functioning of frontline healthcare workers. Especially, sleep quality was focused. This study aimed to investigate the sleep quality of frontline healthcare workers, risk factors for sleep quality, and the effect of Tai Chi training.

**Methods:**

A total of 98 frontline healthcare workers were recruited, coming from the infection department, fever clinic, laboratory, and medical imaging department in a COVID-19-designated hospital in Shanghai. Of them, 50 participated in a 2-week intervention and were randomized to receive a Tai Chi training or relaxation training. Participants were assessed at baseline, 7 and 14 days after participation. Demographic information, sleep quality, and anxiety were measured by using the demographic questionnaire, Pittsburgh Sleep Quality Index (PSQI) and Beck Anxiety Inventory (BAI).

**Results:**

13.3% participants were above the cut-off score (>10) for the PSQI. Regression analysis showed gender, age, working years, and job category had effect on sleep quality. Compared to the control group, participants in the Tai Chi training group had lower scores on both PSQI (*p* < 0.05) and BAI (*p* < 0.01) after the 2-week intervention.

**Conclusion:**

It was demonstrated that poor sleep quality existed in the frontline healthcare workers, which was related to gender, age, working years, and job category. Tai Chi training can dramatically improve their sleep quality and reduce anxiety symptoms.

## Introduction

Coronavirus disease 2019 (COVID-19) was an unprecedented, large-scale public health emergency causing substantial suffering and deaths. It was officially declared a pandemic by the World Health Organization on 11 March 2020 because of its spread and severity (1). As far as it goes, the transmission of the virus and the lockdown measures not only raised public health concerns but also caused tremendous psychological distress, such as stress, anxiety, and depression. Spending long time at home and worrying about family members had prompted more anxiety and depressive symptoms and posed a challenge to our psychological resilience (2).

To fight against COVID-19, healthcare workers are the ones on the front lines and facing the most dangerous situations, playing a dominant role in this pandemic. However, healthcare workers were exhausted by the risk of infection as well as physical and psychological problem because of the overstretched health systems, shortage of personal protective equipment, and overwork. Previous studies have found that healthcare workers and their households had increased risks of admission with COVID-19, especially patient-facing healthcare workers (3). The risk of mental health problems such as stress, anxiety, depressive symptoms, insomnia, and fear was also higher (4, 5). Also, 41% of hospital staff disrupted sleep/wake cycle after the quarantine (6). A study conducted in China also showed that a large number of Chinese healthcare workers experienced mental health problems during this epidemic, and those healthcare workers working at COVID-19-designated hospitals were more likely to report anxiety symptoms (7). Moreover, staff working in the epidemic area of the hospital in Wuhan had a high prevalence of sleep disturbance (41.8%) (8). So, protecting the mental health and improving sleep quality of these healthcare workers is of great importance. In a study of America, family situation, education, friends, and social support were found relevant to self-reported sleep (9). For healthcare workers, the main factors affecting the sleep quality include workloads, coping style, and whether they had confidence to complete tasks and anxiety (8, 10). Treatments for these problems (such as insomnia and anxiety) usually include relaxation training and cognitive therapy (11). But several other interventions may also benefit people with these problems.

With a long history, Tai Chi originated in China and was developed by the famous martial artist Chen Wang-Ting at the end of Ming Dynasty (17th-century A.D.) (12). Tai Chi was usually described as a meditative or internal martial art. It comprehensively incorporated breathing and meditative techniques, body awareness, and imagery (13). In the past decades, a large number of studies have been conducted to explore the advantages of Tai Chi and have proved that Tai Chi has multiple positive effects on our health. Several studies have reported that Tai Chi improves sleep quality and reduces anxiety and depression in both healthy adults and elders (14–16). However, randomized controlled trials remain insufficient in this area, and most participants in these studies are patients and elders.

Thus, this study aimed at investigating sleep quality and its risk factors in frontline healthcare workers and sought to explore the effects of Tai Chi training in improving sleep quality and anxiety symptoms. Based on prior studies, we hypothesized that the frontline healthcare workers receiving Tai Chi training would demonstrate improvements in sleep quality and reduced anxiety compared with the control group.

## Materials and Methods

### Participants

A total of 127 frontline healthcare workers were recruited during 26 December 2020 to 4 January 2021. They all came from the infection department, fever clinic, and laboratory and medical imaging department of a COVID-19-designated hospital in Shanghai, such as doctors, nurses, and nursing staff who can come into contact with patients or test samples of patients’ blood and urine. Inclusion criteria for the study were (1) regular staff of the infection department, fever clinic, laboratory, and medical imaging department; (2) voluntary participation in the survey; (3) understanding the question literally; and (4) among 18–60 years. Exclusion criteria were (1) response time <2 min or >30 min, (2) mental disorders, (3) cognitive impairment, (4) dyslexia, and (5) physical mobility impairment. After screening, 98 participants were included in the data analysis (for participants’ characteristics, see Table 1). As for the intervention, 45 people showed no obvious intention to participate due to personal reasons and 3 people could not use video apps, so the remaining 50 were selected.

**TABLE 1:** One-way ANOVA for the effect of each demographic variable on sleep quality.

Variables	*N* (%)	PSQI			
		Mean	SD	*F*	*p*
**Gender**	98	6.449	3.398	0.149	0.701
Male	16 (16.3)	6.750	2.696		
Female	82 (83.7)	6.390	3.530		
**Age**	98	6.449	3.398	1.620	0.162
≤25	23 (23.5)	6.174	2.871		
26–30	29 (29.6)	5.483	3.511		
31–35	10 (10.2)	7.700	1.703		
36–40	16 (16.3)	8.125	3.384		
41–45	9 (9.2)	6.222	4.466		
≥46	11 (11.2)	6.182	3.816		
**Working years**	98	6.449	3.398	2.572*	**0.043**
≤5	38 (38.8)	6.263	3.020		
6–10	24 (24.5)	5.708	3.303		
11–15	11 (11.2)	7.727	3.197		
16–20	13 (13.3)	8.538	3.777		
≥21	12 (12.2)	5.083	3.655		
**Marital status**	98	6.449	3.398	1.505	0.227
Single	43 (43.9)	6.186	3.034		
Married	54 (55.1)	6.556	3.632		
Divorced	1 (1.0)	12.000			
**Number of children**	98	6.449	3.398	1.878	0.158
None	46 (46.9)	6.065	2.992		
1	43 (43.9)	6.442	3.788		
≥2	9 (9.2)	8.444	3.005		
**Living situation**	98	6.449	3.398	0.247	0.782
With family number	59 (60.2)	6.593	3.620		
Co-living	31 (31.6)	6.097	3.239		
Alone	8 (8.2)	6.750	2.375		
**Job category**	95	6.337	3.331	0.699	0.500
Nurse	72 (75.8)	6.500	3.608		
Doctor	20 (21.1)	6.050	2.188		
Hospital assistant	3 (3.2)	4.333	2.517		
**Educational status**	95	6.337	3.331	0.239	0.788
Associate degree or below	25 (26.3)	5.960	3.234		
Bachelor degree	49 (51.6)	6.531	3.731		
Master degree or above	21 (22.1)	6.333	2.436		

PSQI, Pittsburgh Sleep Quality Index. *p < 0.05.

*The bold values means there are significant differences.*

### Procedure

**FIGURE 1:**
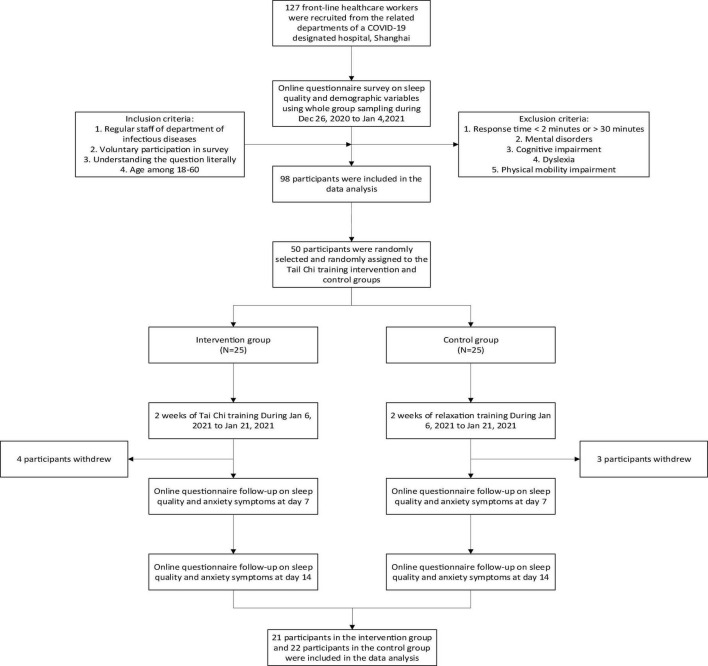
Flow chart of study procedure.

All participants received an online questionnaire. Of the 50 participants who were selected, 25 had been randomly assigned to the intervention group (Tai Chi training) and 25 to the control group (relaxation training). A total of seven participants dropped out of the experiment (four in the intervention group and three in the control group) (see Figure 1).

Both Tai Chi training and relaxation training lasted 2 weeks during 6 January 2021 to 21 January 2021. Tai Chi training was conducted by a professional 24-style Tai Chi teacher. The trainer formulated the teaching outline, teaching contents, methods, and schedule. All participants in this group received a Tai Chi course with 6 pretraining sessions within 3 days before the intervention. In the process of the course, participants were asked to practice movement, posture, and movement speed, until they learned the correct posture. After each pretraining session, each participant played Tai Chi videos online to facilitate self-practice. In the last pretraining session, a Tai Chi exam was setup. Only after passing the exam can participants attend the formal intervention, and all participants passed it at last. During the 2-week intervention, the participants practiced Tai Chi with the teacher and teaching video from 10:00 to 10:30 every day. While the participants in control group received a relaxation training using elastic belts, hula hoops, skipping rope, and pushups. They all did free exercise from 10:00 to 10:30 every day. All participants opened Tencent conference software when doing training to ensure that the training intensity and duration were consistent in both groups in order to reduce the error. All participants signed informed consent form.

### Measures

The demographic information was collected at baseline, such as age, working years, number of children, educational status, and so on. Additionally, two self-report questionnaires were also administered at the baseline, 7 and 14 days after intervention started. Sleep quality was measured by the Pittsburgh Sleep Quality Index (PSQI) (17). The PSQI consists of 4 items to measure subjects’ sleep quality, with higher scores indicating worse sleep quality (0–5: sleep quality is very good; 6–10: sleep quality is good; 11–15: sleep quality is bad; 16–21: sleep quality is very bad). The Cronbach’s α in this study was 0.77. Anxiety symptoms were measured by the Beck Anxiety Inventory (BAI) (18). The BAI is a widely used self-reported measure of anxiety symptoms consisting of 21 self-rated questions with scores ranging from 0 to 3. Total scores range from 0 to 63, with a higher score indicating a higher level of anxiety symptoms. For this study, Cronbach’s alpha coefficient was 0.89.

### Statistical Analysis

An analysis of variance (ANOVA) and an ordinary least squares (OLS) regression analysis were computed to test related risk factors. The OLS regression is commonly used to apply for measuring the relationship between dependent variable and more than one independent variable. Independent variables in this study were those demographic variables. Then, to compare the demographic variables of participants in the two groups (Tai Chi training, relaxation training), we computed a χ^2^ test. Finally, we compared the difference in sleep quality and anxiety symptoms with time points (baseline, 7 and 14 days) between the intervention group and the control group.

## Results

### Sleep Quality of Frontline Healthcare Workers

In all of the participants (mean age = 33.04, SD = 9.02), 82 were women (83.7%). Of all participants, 43 (43.9%) reported lower than 5 scores for PSQI and 42 (42.9%) reported 6–10 scores. While the rest 13 (13.3%) reported over 10 scores for PSQI, which meant that their sleep quality was bad during the epidemic. These results showed that sleep quality of frontline healthcare workers was affected by the COVID-19 pandemic and over 10% of them experienced declines in sleep quality.

### The Influence Factors of Sleep Quality

The one-way ANOVA revealed a significant effect for working years [*F*_(4_,_95)_ = 2.572, *p* = 0.043]. No significant difference was observed in other demographic variables (Table 1).

The result of regression analysis showed significant effects for gender (β = –0.317, *p* = 0.032), age (β = 1.187, *p* = 0.014), working years (β = –1.139, *p* = 0.009), and job category (β = –0.611, *p* < 0.001) (Table 2). There was no significant difference in other variables. These variables account for 9.2% of the total variation (adjusted *R*^2^ = 0.092). We can see in this study that gender, working years, and job category were found to have a negative relationship, but age was found to have a positive relationship with sleep quality. For higher scores of PSQI representing worse sleep quality, these results suggested that better sleep quality was related to younger age and longer working years.

**TABLE 2:** OLS regression analysis of the effect of demographic variables on sleep quality.

Model	Variables	B	β	*t*	*p*	*R* ^2^	Adjusted *R*^2^	*F*	*p*
**PSQI**						0.171	0.092	2.163	**0.039**
	Gender	–2.881	–0.317	−2.181*	**0.032**				
	Age	0.448	1.187	2.501*	**0.14**				
	Working years	–0.434	–1.139	−2.670**	**0.009**				
	Marital status	–0.512	–0.079	–0.409	0.684				
	Number of children	1.483	0.290	1.649	0.103				
	Living situation	0.175	0.033	0.232	0.817				
	Job	–3.959	–0.611	−3.563***	** < 0.001**				
	Educational status	0.081	0.017	0.141	0.888				

*PSQI, Pittsburgh Sleep Quality Index.*

*p < 0.05; **p < 0.01; ***p < 0.001.

*The bold values means there are significant differences.*

### The Difference in Sleep Quality and Anxiety Symptoms After Intervention

Among all the participants, demographic variables in the intervention group and control group were examined using χ^2^ tests (Table 3). The results revealed no significant difference in all demographic variables at baseline.

**TABLE 3:** Demographic variables of participants in the intervention and control groups in the Tai Chi training study.

Variables	*N* (%)			χ^2^	*p*
	Summary	Intervention group	Control group		
**Gender**	43	21	22	0.410	0.522
Male	3 (7.0)	2 (9.5)	1 (4.5)		
Female	40 (93.0)	19 (90.5)	21 (95.5)		
**Age**	43	21	22	5.895	0.317
≤25	22 (51.2)	10 (47.6)	12 (54.5)		
26–30	10 (23.3)	6 (28.6)	4 (18.2)		
31–35	3 (7.0)	0	3 (13.6)		
36–40	3 (7.0)	1 (4.8)	2 (9.1)		
41–45	4 (9.3)	3 (14.3)	1 (4.5)		
≥46	1 (2.3)	1 (4.8)	0		
**Working years**	43	21	22	4.312	0.505
≤5	28 (65.1)	14 (66.7)	14 (63.6)		
6–10	5 (11.6)	1 (4.8)	4 (18.2)		
11–15	1 (2.3)	1 (4.8)	0		
16–20	5 (11.6)	3 (14.3)	2 (9.1)		
≥21	4 (9.3)	2 (9.5)	2 (9.1)		
**Marital status**	43	21	22	0.043	0.835
Single	28 (65.1)	14 (66.7)	14 (63.6)		
Married	15 (34.9)	7 (33.3)	8 (36.4)		
**Number of children**	43	21	22	1.111	0.774
None	30 (69.8)	14 (66.7)	16 (72.7)		
1	10 (23.3)	5 (23.8)	5 (22.7)		
≥2	3 (7.0)	2 (9.5)	1 (4.5)		
**Living situation**	43	21	22	2.355	0.308
With family number	17 (39.5)	7 (33.3)	10 (45.5)		
Co-living	21 (48.8)	10 (47.6)	11 (50.0)		
Alone	5 (11.6)	4 (19.0)	1 (4.5)		

**FIGURE 2:**
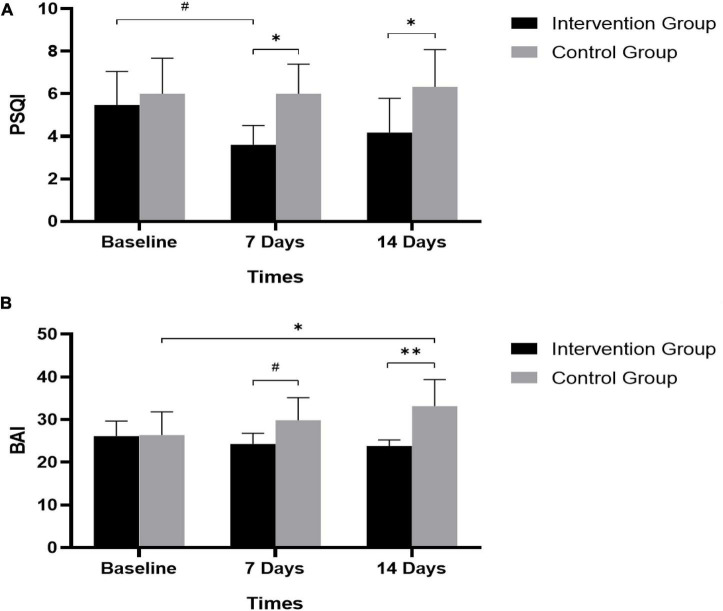
Effectiveness of Tai Chi training in improving sleep quality and reducing anxiety symptoms. **(A)** PSQI scores in intervention and control group, *p < 0.05; #*p* = 0.077. **(B)** BAI scores in intervention and control group, *p < 0.05; **p < 0.01; #*p* = 0.071. PSQI, Pittsburgh Sleep Quality Index; BAI, Beck Anxiety Inventory.

Then, we compared the PSQI and BAI scores between the intervention group and control group to figure out whether there is a difference in the effect of Tai Chi. Table 4 shows the results. Before the intervention, both groups did not differ in their PSQI scores [Tai Chi = 5.476, SD = 3.459; control = 6.000, SD = 3.792; *F*_(1_,_41)_ = 0.260, *p* = 0.611] or BAI scores [Tai Chi = 26.143, SD = 7.683; control = 26.409, SD = 12.192; *F*_(1_,_41)_ = 0.008, *p* = 0.929]. At the time of 7 and 14 days, the intervention group reported lower PSQI scores than the control group [7 days: *F*_(1_,_41)_ = 5.204, *p* = 0.024; 14 days: *F*_(1_,_41)_ = 4.386, *p* = 0.038]. As for BAI scores, the results showed significant difference between the two groups at the time of 7 days [*F*_(1_,_41)_ = 9.786, *p* = 0.002] but no significant difference at the time of 14 days. Changes in PSQI and BAI scores over time differed between the two groups (see Figure 2). That is to say, the intervention group had better sleep quality and fewer anxiety symptoms compared to the control group.

**TABLE 4:** The difference in sleep quality and anxiety symptoms between the intervention and control groups.

Model	Time	Intervention group			Control group			*F*	*p*	η^2^
		*N*	Mean	SD	*N*	Mean	SD			
**PSQI**		63	4.429	3.176	64	6.109	3.533	8.006**	**0.005**	0.062
	Baseline	21	5.476	3.459	22	6.000	3.792	0.260	0.611	0.002
	7 days	20	3.600	1.957	21	6.000	3.066	5.204*	**0.024**	0.041
	14 days	22	4.182	3.621	21	6.333	3.838	4.386*	**0.038**	0.035
**BAI**		63	24.746	5.697	64	29.750	12.655	8.471**	**0.004**	0.065
	Baseline	21	26.143	7.683	22	26.409	12.192	0.008	0.929	0.000
	7 days	20	24.300	5.411	21	29.857	11.642	3.314	0.071	0.027
	14 days	22	23.818	3.172	21	33.143	13.731	9.786**	**0.002**	0.075

*PSQI, Pittsburgh Sleep Quality Index; BAI, Beck Anxiety Inventory.*

*p < 0.05; **p < 0.01.

*The bold values means there are significant differences.*

## Discussion

This study investigated sleep quality and its factors in frontline healthcare workers and the effect of Tai Chi training from a randomized controlled trial. The factors affecting sleep quality were tested, and sleep quality and anxiety symptoms were compared between the Tai Chi training group and the relaxation control group. Over 10% frontline healthcare workers experienced bad sleep quality. Gender, age, working years, and job category were found to have effect on sleep quality. Additionally, compared to control group, Tai Chi training was associated with greater reductions in both PSQI and BAI scores during the 2-week intervention. It was suggested that Tai Chi training can improve sleep quality and reduce anxiety symptoms, and it may work better than traditional relaxation training.

Several studies indicated that over 40% of hospital staff reported sleep disturbance during the pandemic (8). It was suggested that 11.3% participants had bad sleep quality in this study, which was lower than previous results. This could be due to the time of this study, which was conducted in January, 2021 when domestic epidemic tended to be stable. The situation of overstretched health systems has eased and works of frontline healthcare workers became more regular at that time. In addition, several existing studies have identified a few factors to be associated with sleep quality, including female, older age, divorced or separated marital status, lower educational level, and socioeconomic status (19–21). Results of this study demonstrated that worse sleep quality was related to older age, which was consistent with previous studies (22). Possible reasons may include supporting children, taking care of parents, and a higher risk of medical conditions. We also found that longer working years were associated with better sleep quality. This may be caused by the proficiency or higher position of people with longer working years. Moreover, for healthcare workers, due to rotating working hours and night work, their circadian rhythm can be easily disrupted and result in impaired sleep (23). In keeping with previous findings, nurses were more likely to have bad sleep quality compared to hospital assistants or doctors because of their longer working hours, more night shifts, and heavy workload, especially during the pandemic. Besides, designated hospitals implemented a rotation system (working for 14 days and then following isolation of 14 days) during the epidemic. The greater intensity and longer hours of work than normal situation brought more pressure to frontline healthcare workers and influenced their sleep quality. So, interventions are really needed to improve sleep quality of frontline healthcare workers, particularly nurses. There were no significant differences in marital status or educational status, which need to be investigated further with a bigger sample.

Previous studies focused on patients and elders have explored the effect of Tai Chi on mental health and found that Tai Chi played a role in improving sleep quality and anxiety symptoms (24, 25). It should be noted that Tai Chi training can also improve frontline healthcare workers’ sleep quality and anxiety symptoms that was proved in this study, expanding the applicability of Tai Chi effects in the crowd. Another point worth mentioning is that many studies showed that Tai Chi was helpful to people’s mental health, but most of them compared it with a blank control group or could not figure out the differences between different methods (26, 27). This study compared the effect of Tai Chi training and relaxation training, indicating that the effect of Tai Chi training in improving sleep quality and anxiety symptoms is probably better than relaxation training. Tai Chi, which is not equivalent to random relaxation training, is a full set of punches and gradually increases the intensity during the training. It is inherently a complex intervention composed of multiple components such as breathing, mindfulness, imagery, and psychosocial interactions (12). Each of these components has potentially independent and synergistic therapeutic value. When practicing, our movement, breathing, and thought work together, promoting the function of human nervous system and coordinating the excitatory and inhibitory processes of the nervous system. All of these showed that Tai Chi training has its own irreplaceable advantages.

Although important conclusions were reached, some limitations still deserved discussion. First, unbalanced gender in the sample. Due to female participants accounting for 93% of the total participants, results of gender need to be discussed carefully. Second, after the experiment ended, no follow-up was performed. How long the training effect lasts is still unclear. Further studies are needed. Finally, Tai Chi has many styles, only one of them was trained in this study. More Tai Chi styles can be put into for comparison in future research.

## Conclusion

This study demonstrates that poor sleep quality existed in the frontline healthcare workers, which was related to gender, age, working years, and job category. Additionally, Tai Chi training has an effect on improving sleep quality and reducing anxiety symptoms in frontline healthcare workers. Follow-up study and different styles of Tai Chi need more research in the future.

## Data Availability Statement

The original contributions presented in the study are included in the article/supplementary material, further inquiries can be directed to the corresponding author/s.

## Ethics Statement

The studies involving human participants were reviewed and approved by the Ethics Committees of Naval Medical University. The patients/participants provided their written informed consent to participate in this study.

## Author Contributions

JZ, KY, and ZS contributed to the writing of the manuscript and statistical analysis of the manuscript. WL, CY, and LW led the whole study and carried out the study. XL, LB, and XW performed the investigation and collection of all data and part of the statistical analysis of the manuscript. All authors contributed to the article and approved the submitted version.

## Conflict of Interest

The authors declare that the research was conducted in the absence of any commercial or financial relationships that could be construed as a potential conflict of interest.

## Publisher’s Note

All claims expressed in this article are solely those of the authors and do not necessarily represent those of their affiliated organizations, or those of the publisher, the editors and the reviewers. Any product that may be evaluated in this article, or claim that may be made by its manufacturer, is not guaranteed or endorsed by the publisher.
